# Supplementary material for: Glucagon-like Peptide-1 Receptor in the Human Hypothalamus Is Associated with Body Mass Index and Colocalizes with the Anorexigenic Neuropeptide Nucleobindin-2/Nesfatin-1
Source: Int J Mol Sci. 2022 Nov 28;23(23):14899. doi: 10.3390/ijms232314899 (PMC9740138; doi:10.3390/ijms232314899)
Supplement: Supplementary file 1 [file ijms-23-14899-s001.zip › Supplementary Table S1.pdf]

Supplementary Table S1: Distribution of GLP1R protein expression in the human hypothalamus of all examined cases, presented as immunostaining score, using rabbit polyclonal anti-GLP-1R antibody (Origene, Rockville, MD, USA, TA336864).

**Case**	**PVN**	**SON**	**IFN**	**LH**	**PeF**	**VMH**	**DMH**	**TM**	**MPO**	**SCh**	**MN**	**BN**	**DB**	**BST**
1	++	+++	++	++	++	++	+	++	+	−	++	+++	++	++
2	+++	+++	++	+++	+++	++	++	+	++	+	−	+++	++	++
3	++	+++	+	++	+	−	−	+	++	+	+++	+++	+++	++
4	++	+++	++	+++	+++	+	++	+	+++	+	++	++	++	++
5	++	++	+	+	+	−	−	+	−	+	+	++	n/a	−
6	+++	+++	++	+++	+++	+	++	++	++	−	++	+++	+++	++
7	++	++	+	+	+	−	−	+	−	+	+	++	++	n/a
8	++	+++	+	++	++	+	+	++	−	−	+	++	+++	++
9	++	+++	+++	+++	++	++	++	++	++	++	−	++	++	++
10	++	+++	++	++	++	+	+	++	+	n/a	++	++	++	++
11	++	++	+++	+++	++	+	++	+	++	++	++	++	+	+++
12	+++	++	–	++	++	++	++	++	n/a	−	−	+++	++	+++
13	++	++	++	+	−	n/a	−	−	+	n/a	++	+	+	n/a
14	+++	+++	+++	+++	+	+	+	+	−	+	++	++	++	+++
15	+	++	n/a	++	+	n/a	−	−	+	−	+++	+++	++	n/a
16	++	+++	++	++	++	++	−	−	++	−	−	++	+++	+++
17	+++	+++	+++	+	+	+++	++	++	++	−	++	++	n/a	n/a
18	+++	+++	+	+	+	+	+	+	++	−	++	+++	+++	++

19	++	++	+	++	++	−	+	++	−	++	+++	++	++	++
20	++	+++	++	+	++	+	n/a	n/a	++	−	++	++	++	++
21	++	+++	+	+	+	−	−	+	+	+	+	+++	++	++
22	++	+++	+	+	++	+	+	++	++	−	++	++	++	++
23	+++	+++	++	++	+	++	+	++	++	+	−	++	+++	++
24	++	++	+	++	+	++	−	n/a	−	−	+	++	++	n/a
25	++	++	++	++	+	++	n/a	+	+	+	−	++	+	++
26	+	++	+++	++	++	++	++	++	++	−	+	n/a	++	++
27	++	++	n/a	+	+	−	+	++	+	−	+++	++	++	n/a
28	++	+++	+	+	++	+	+	+	+	+	+	++	n/a	+
**Total**														
+++	7/28	17/28	5/28	6/28	3/28	1/28	0/28	0/28	1/28	0/28	4/28	8/28	6/28	4/28
++	19/28	11/28	10/28	12/28	12/28	9/28	8/28	12/28	12/28	3/28	11/28	18/28	16/28	16/28
+	2/28	0/28	10/28	10/28	12/28	10/28	10/28	11/28	8/28	10/28	7/28	1/28	3/28	1/28
−	0/28	0/28	1/28	0/28	1/28	6/28	8/28	3/28	6/28	13/28	6/28	0/28	0/28	1/28

Abbreviations: BN, basal nucleus; BST, bed nucleus of the stria terminalis; DB, diagonal band; DMH, dorsomedial hypothalamic nucleus; IFN, infundibular nucleus; LH, lateral hypothalamic area; MN, mammillary nucleus; MPO, medial preoptic nucleus; PeF, perifornical nucleus; PVN, paraventricular nucleus; SCh, suprachiasmatic nucleus; SON, supraoptic nucleus; TM, tuberomammillary nucleus; VMH; ventromedial hypothalamic nucleus.

+++, >50% of cells exhibit high immunoexpression; ++, >50% of cells exhibit moderate immunoexpression; +, >50% of cells exhibit low immunoexpression; −, no staining; n/a; not assessed.
